# Increasing reports of non-tuberculous mycobacteria in England, Wales and Northern Ireland, 1995-2006

**DOI:** 10.1186/1471-2458-10-612

**Published:** 2010-10-15

**Authors:** Jonathan E Moore, Michelle E Kruijshaar, L Peter Ormerod, Francis Drobniewski, Ibrahim Abubakar

**Affiliations:** 1 Health Protection Agency Centre for Infections, Respiratory Diseases Department - Tuberculosis Section, 61 Colindale Avenue, London, NW9 5EQ, UK; 2 Royal Blackburn Hospital, Department of Chest Medicine, Haslingden Road, Blackburn, Lancashire, BB2 3HH, UK; 3 Health Protection Agency Centre for Infections, National Mycobacterium Reference Laboratory, Abernethy Building, Institute of Cell and Molecular Science (ICMS), 2 Newark Street, London, E1 2AT, UK

## Abstract

**Background:**

Non-tuberculous mycobacteria have long been identified as capable of causing human disease and the number at risk, due to immune-suppression, is rising. Several reports have suggested incidence to be increasing, yet routine surveillance-based evidence is lacking. We investigated recent trends in, and the epidemiology of, non-tuberculous mycobacterial infections in England, Wales and Northern Ireland, 1995-2006.

**Methods:**

Hospital laboratories voluntarily report non-tuberculous mycobacterial infections to the Health Protection Agency Centre for Infections. Details reported include age and sex of the patient, species, specimen type and source laboratory. All reports were analysed.

**Results:**

The rate of non-tuberculous mycobacteria reports rose from 0.9 per 100,000 population in 1995 to 2.9 per 100,000 in 2006 (1608 reports). Increases were mainly in pulmonary specimens and people aged 60+ years. The most commonly reported species was *Mycobacterium avium-intracellulare *(43%); *M. malmoense *and *M. kansasii *were also commonly reported. *M. gordonae *showed the biggest increase over the study period rising from one report in 1995 to 153 in 2006. Clinical information was rarely reported.

**Conclusions:**

The number and rate of reports increased considerably between 1995 and 2006, primarily in older age groups and pulmonary specimens. Increases in some species are likely to be artefacts but real changes in more pathogenic species, some of which will require clinical care, should not be excluded. Enhanced surveillance is needed to understand the true epidemiology of these infections and their impact on human health.

## Background

The non-tuberculous mycobacteria (NTM), mycobacteria other than members of the *Mycobacterium tuberculosis *complex and *M. leprae*, have been documented since the 1950s as organisms capable of causing human disease [1], receiving greater clinical recognition as the incidence of tuberculosis fell [2]. Over 100 species of NTM have been identified but only around 15 are considered pathogenic in humans [3]. While NTM can cause pulmonary, lymph node, skin and disseminated disease in humans, many species are found ubiquitously in environmental reservoirs and in various domestic and wild animals and are therefore frequently isolated from clinical samples due to contamination. In general, NTM only cause disease in immunocompromised individuals, which is relative in small children, or actual, e.g. in those with HIV infection or structural tissue damage in the lungs due to chronic obstructive pulmonary disease (COPD), cystic fibrosis, or scarring due to a number of conditions including tuberculosis. These infections can be difficult to treat, are often reported as having in-vitro resistance on single antibiotic testing, although synergy with combinations has been shown [4], and can complicate the diagnosis of tuberculosis. NTM infections are not normally transmissible between humans.

Determining the clinical relevance of NTM isolates can be difficult and can depend on a combination of the site from where the sample was taken, the number of isolations, and the species involved, as well as clinical symptoms and any predisposing conditions of the patient [5]. *M. avium-intracellulare *often presents as disseminated NTM infection in AIDS patients [6], but cervical lymph node disease in young children and pulmonary disease mimicking tuberculosis in COPD patients. *M. kansasii *is also commonly associated with pulmonary infections closely resembling tuberculosis, both clinically and radiologically, and may be misdiagnosed [7]. *M. malmoense *may be clinically significant regardless of the site of infection [8] which is usually pulmonary, again mimicking tuberculosis, as does *M. xenopi*. *M. abscessus *has been described as a prevalent NTM infection in children with cystic fibrosis [9]. *M. gordonae *is normally considered non-pathogenic and is often found as a laboratory contaminant but does sometimes cause disease, even in immunocompetent patients [10].

The most recent study of national surveillance data for England and Wales by Lamden *et al*. reported an increase in NTM reports from 123 in 1982 to 392 in 1994 [11]. Most of this increase was attributed to an increase in *M. avium-intracellulare *infections, notably in the form of disseminated disease and in males aged 25 to 44 years. Other NTM infections in this study were most commonly reported in those aged 45 and over. Prior to the increases in HIV infection and AIDS, most NTM infections were found in those aged 50-59 with pre-existing lung conditions or occupational exposure to dusts [12].

Several reports from other western countries have suggested the incidence of NTM disease is increasing [12-14] but there is currently a lack of routine, national surveillance based evidence to support this [12]. The number at risk of NTM infection due to immune-suppression is rising owing to factors such as more HIV diagnoses, new treatments for pre-existing conditions such as cystic fibrosis and malignancies, and an increasingly elderly population with significant levels of COPD and structural lung disease. This study investigated recent trends in, and described the epidemiology of, NTM infections in England, Wales and Northern Ireland between 1995 and 2006.

## Methods

Hospital laboratories in England, Wales and Northern Ireland voluntarily report mycobacterial infections, as part of routine reporting of all infectious agents, to the Health Protection Agency (HPA) Centre for Infections. Patient samples identified by local hospital laboratories as positive for mycobacteria are forwarded to specialist reference laboratories that will identify the species and report back to the local laboratory. All isolates deemed clinically significant by the source hospital are reported to the HPA. Details routinely reported include age and sex of the patient, species, specimen type and source laboratory. All reports of NTM between 1995 and 2006 were analysed.

Incidence rates and percentage change were calculated for each species of NTM reported. Demographic information was analysed for all NTM as a whole, with further detailed analyses for the three most commonly reported species and the species showing the biggest percentage increase over the study period. Clinical information in comments fields was also reviewed for these species to assess the clinical significance of reports and consider associated risk factors for infection.

Office for National Statistics mid-year population estimates were used for the calculation of rates of NTM infection per 100,000 population. Analyses by region were completed based on the region of the reporting source laboratory. Site of disease was inferred from specimen type; as in some cases NTM were isolated from more than one site, the total number of reports by site may exceed the total numbers reported otherwise.

The Health Protection Agency has Patient Information Advisory Group approval to hold and analyse national surveillance data for public health purposes under Section 60 of the Health and Social Care Act 2001.

## Results

**Figure 1:**
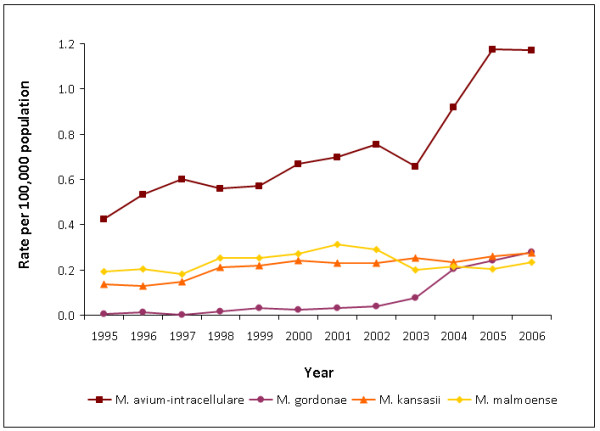
**Rates of non-tuberculous mycobacteria reports (selected species), England, Wales and Northern Ireland, 1995-2006**.

The rate of all NTM reports increased from 0.9 per 100,000 population in 1995 to 2.9 per 100,000 in 2006 (Table 1). Except from 2002 to 2003, numbers increased annually, rising to 1608 reports in 2006, a 250% increase compared to 1995. The most commonly reported species was *M. avium-intracellulare*, accounting for 43% of all reports, followed by *M. malmoense *(14%) and *M. kansasii *(13%); *M. gordonae *showed the biggest percentage increase in reports over the study period. Changes in rates for these four species are shown in Figure 1. *M. abscessus *was not reported prior to 2000, *M. peregrinum *was not reported before 2004, and an increasing number of isolates were reported without a species name.

**Table 1:** Non-tuberculous mycobacteria reports and rates by species, England, Wales and Northern Ireland, 1995, 2000, 2006

	1995		2000		2006		
		
Species							Total number (1995-2006)
	Number	Rate per 100,000	Number	Rate per 100,000	Number	Rate per 100,000	
*M. avium-intracellulare*	225	0.4	360	0.7	649	1.2	4732
*M. malmoense*	101	0.2	145	0.3	130	0.2	1510
*M. kansasii*	71	0.1	129	0.2	152	0.3	1380
*M. xenopi*	33	0.1	50	0.1	117	0.2	827
*M. chelonae*	5	0.0	42	0.1	117	0.2	708
*M. fortuitum*	6	0.0	27	0.1	90	0.2	518
*M. gordonae*	1	0.0	12	0.0	153	0.3	516
*M. marinum*	14	0.0	27	0.1	27	0.0	274
*M. abscessus*	0	0.0	1	0.0	60	0.1	172
*M. peregrinum*	0	0.0	0	0.0	30	0.1	69
*M. terrae*	0	0.0	2	0.0	4	0.0	33
*M. szulgai*	1	0.0	0	0.0	1	0.0	30
*M. fortuitum/chelonae *group	0	0.0	0	0.0	4	0.0	23
*M. scrofulaceum*	0	0.0	1	0.0	1	0.0	15
*M. flavescens*	0	0.0	0	0.0	0	0.0	1
Unnamed species	2	0.0	5	0.0	73	0.1	231

Total	459	0.9	801	1.5	1608	2.9	11039

Most NTM reports were in males (6518/10863, 60%) and the rate of reports was consistently higher each year in males compared to females (3.3 v 2.4 per 100,000 population in 2006). The majority of reports were from people aged 60 years or over (5717/10824, 53%). The greatest numbers of reports were from the North East (2126/11039, 19%) and London (1972/11039, 18%); the highest rate of reports in 2006 was from the North East (10.3 per 100,000) followed by Northern Ireland and London (4.9 and 4.4 per 100,000 respectively). The biggest increases in reports from 1995 to 2006 were in Northern Ireland (1129%) and the North East (1100%). Eighty-one percent (8832/10895) of reports were from a pulmonary site. However, this varied with age: in those aged under 15, 83% (423/507) of reports were extra-pulmonary.

### M. avium-intracellulare

**Figure 2:**
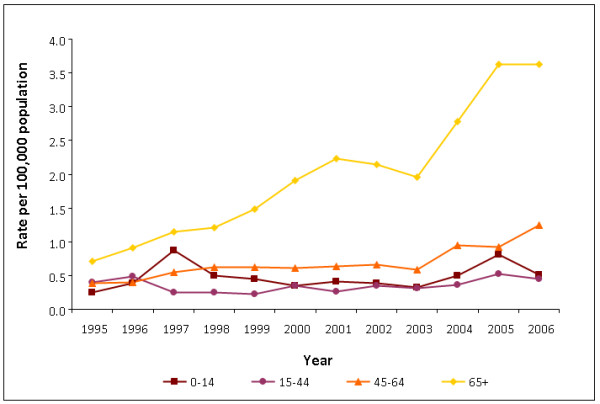
**Rates of *M. avium-intracellulare *reports by age group (years), England, Wales and Northern Ireland, 1995-2006**.

The rate of reports of *M. avium-intracellulare *rose three-fold from 0.4 per 100,000 in 1995 to 1.2 per 100,000 in 2006 (Table 1, Figure 1). Increases were seen in both sexes, but mostly in people aged 65 and over (from 0.7 per 100,000 to 3.6 per 100,000 - Figure 2). The increase was also more pronounced in pulmonary sites, rising from 45% of reports (113/251) in 1995 to 80% (603/757) in 2006. The percentage of reports from blood specimens declined from 28% (71/251) to 3% (21/757) in the same period.

Useful clinical comments were available for just 8% (363/4732) of reports. Of these, HIV/AIDS, chronic respiratory illness, cystic fibrosis and cancer were mentioned in 30% (109), 21% (75), 11% (39) and 5% (18) of reports respectively.

### M. malmoense

*M. malmoense *was the second most commonly reported species over the study period (Table 1). The rate of reports was stable at between 0.2 and 0.3 per 100,000 (Figure 1), a mean of 126 per year. The majority of reports were in men (939/1488, 63%) and in people aged 45 years and over (1255/1480, 85%); just 5% (77) occurred in young adults aged 15-44 years and 10% (148) were in those under 15 years. Ninety percent (1266/1404) of infections were from pulmonary sites. Where information was available in clinical comments (5%, 73/1510), chronic respiratory illness was most commonly reported (24, 33%), followed by cancer (4, 5%).

### M. kansasii

The rate of *M. kansasii *reports rose from 0.1 per 100,000 in 1995 to 0.3 per 100,000 in 2005 (Table 1, Figure 1). Increases were seen in both sexes, but mainly in those aged 45 and over. Ninety-two percent (1282/1389) of reports were from a pulmonary site. Chronic respiratory illness was again the most commonly reported clinical comment (20/69, 29%) where information was available (69/1380, 5%).

### M. gordonae

Reports of *M. gordonae *increased from just 1 in 1995 to 153 in 2006, when it was the second most commonly reported species and at a rate of 0.3 per 100,000 population (Table 1, Figure 1). In contrast to the trends seen by region for all NTM, reports were most commonly from Yorkshire and the Humber (133/516, 26%), with just 0.4% (2/516) coming from the North East. Sixty-one percent of reports were in males (307/505). Although the increase was largest in those over 65 years old (rising to 0.8 per 100,000 in 2006) and in pulmonary sites, numbers increased in both sexes, across all age groups and in both pulmonary and extra-pulmonary infections. Where clinical comments were present (41/516, 8%), 49% (20/41) mentioned chronic respiratory illnesses.

## Discussion

### Main findings

The number and rate of NTM reports increased considerably between 1995 and 2006, continuing the trend reported previously [11]. Increases were seen across many species reported, but were most noticeable in *M. avium-intracellulare *and *M. gordonae*. The rise in all NTM was predominantly in pulmonary sites in those over 60 years of age.

Sixty percent of all NTM reports were in men and rates were consistently higher in men across all years. Men may be more susceptible to NTM infection due to higher historical rates of smoking and COPD. A higher index of suspicion of tuberculosis in men, and therefore an increased submission of investigative samples from men, may also have contributed to this finding if NTM were found as a consequence.

Previous increases in *M. avium-intracellulare *infections have been linked to increases in HIV/AIDS [6]. However, the introduction of highly active anti-retroviral therapy (HAART) in the mid-1990s has since led to greater immune-competence in these patients. The increases observed here were in older age groups and mainly from pulmonary sites, inconsistent with disseminated infection in HIV/AIDS patients; the percentage of reports from blood specimens also fell considerably. The changes seen may therefore be associated with increases in immune-suppressive therapies for other conditions in older age groups.

Ninety-two per cent of *M. kansasii *reports were from pulmonary specimens, which is consistent with the presentation of such infections resembling pulmonary tuberculosis [7]. The increases seen in the number of pulmonary *M. kansasii *reports may have important implications for both patients and public health as treatment and contact tracing for tuberculosis may be inappropriately/unnecessarily initiated. This highlights the need for prompt submission of specimens for identification, and awaiting speciation prior to large-scale contact investigation [15].

### Interpreting the trends

The HPA's reporting guidelines state that only clinically significant isolates should be reported and these criteria did not change over the study period, so a change in guidelines cannot explain the observed trend. It is possible, however, that the guidelines have not been adequately followed and so the increases observed may not reflect clinical significance. The correct reporting of only clinically significant isolates appeared to vary by region and species - just 1% of all *M. gordonae *reports, rarely considered clinically significant, came from the North East, yet the region accounted for the highest proportion of reports of all NTM, suggesting greater adherence to guidelines in this region. The relatively small increase in *M. malmoense *reports, which guidelines state may always be considered clinically significant, appears to indicate that some of the larger increases in other species, where clinical significance is less defined, may be accounted for by increased tendency of laboratories to report non-clinically significant isolates.

In a region of the Netherlands, just 25% (53/212) of all pulmonary NTM isolated met the American Thoracic Society diagnostic criteria for clinical significance based on a review of medical records [12,13], highlighting the scope for over-reporting. However, this did vary by species: just 1/48 (2%) of *M. gordonae *isolates were considered clinically relevant compared to 24/59 (41%) isolates of *M. avium*, for example.

Changes in laboratory techniques may have contributed to increased detection of NTM as the use of automated liquid culture gradually became more widespread over the study period. In the early 1990s, such methods were considered expensive and impractical for many laboratories [16] but by 2000, increasing numbers of laboratories were purchasing such systems [17], and the latest HPA standard operating procedures from 2006 state the use of automated liquid culture as the standard recommendation [18]. Experience of the authors suggests that *M. gordonae *and *M. chelonae *were rarely isolated prior to the use of liquid culture media. Since this change, moderate numbers have been isolated but very few are clinically relevant and are more likely to be contaminants of samples, particularly where the patient has no chest x-ray changes. The large increase in *M. gordonae *reports was not specific to any particular age group, sex or site and is likely to be associated with increased detection and reporting of isolates that are not clinically significant.

The introduction of DNA probe identification methods [17] may have contributed to increasingly specific and accurate speciation. The apparent emergence of some species, for example *M. abscessus*, are due to changes in taxonomy. Other increases seen may be linked to a rise in the number of samples submitted for identification of *M. tuberculosis *as rates and awareness of tuberculosis have increased (rates of tuberculosis in England increased from 11.2 per 100,000 in 1999 to 15.4 per 100,000 in 2006) [19].

### Limitations

Some duplicate reporting by local hospital laboratories and specialist reference laboratories may be occurring, leading to an artefactual rise, especially in the North East. It is also possible that multiple isolations from the same patient were reported and so the numbers of isolates may not reflect numbers of patients. Having said this, multiple isolates should impact only on the numbers and not on the trends observed, assuming the numbers of samples submitted per patient has remained similar.

The lack of clinical data accompanying most reports did not allow us to assess their actual/true clinical significance, and it was therefore not possible to interpret whether trends were related to contamination or colonisation rather than clinical disease.

## Conclusions

Reports of NTM in England, Wales and Northern Ireland continue to increase but the clinical significance of this increase remains unknown. A number of infections inevitably require clinical care and a small proportion may lead to inappropriate treatment/contact investigation if tuberculosis is assumed. However, due to the non-transmissible nature of these infections, they have failed to attract the level of attention or policy action afforded to tuberculosis, and a lack of clinical information in surveillance data limits the interpretation of the observed trends.

While artefactual changes may, at least for some species, have contributed to the observed increase in NTM reports, real changes in the more notable, pathogenic species should not be excluded. Further study of specific reports, involving a detailed assessment of patient notes, is required to validate and understand the data observed. The collection of detailed clinical data, through a dedicated enhanced surveillance system, is needed to aid the understanding of NTM epidemiology and the assessment of the real impact of increasing reports on human health.

## Competing interests

The authors declare that they have no competing interests.

## Authors' contributions

JEM conducted the analyses and wrote the first draft of the manuscript. IA suggested the analysis and, along with MK, contributed to the interpretation of the data and writing of the manuscript. LPO and FD contributed to the interpretation of the data and revision of the manuscript, with particular reference to clinical and laboratory aspects respectively. All authors read and approved the final manuscript.

## Pre-publication history

The pre-publication history for this paper can be accessed here:

http://www.biomedcentral.com/1471-2458/10/612/prepub
